# CMOS-MEMS Test-Key for Extracting Wafer-Level Mechanical Properties

**DOI:** 10.3390/s121217094

**Published:** 2012-12-12

**Authors:** Wan-Chun Chuang, Yuh-Chung Hu, Pei-Zen Chang

**Affiliations:** 1 Department of Mechanical and Electro-Mechanical Engineering, National Sun Yat-sen University, Kaohsiung 80424, Taiwan; 2 Department of Mechanical and Electro-Mechanical Engineering, Center of Green Technology, National ILan University, ILan 260, Taiwan; E-Mail: ychu@niu.edu.tw; 3 Institute of Applied Mechanics, National Taiwan University, Taipei 10617, Taiwan; E-Mail: changpz@ntu.edu.tw

**Keywords:** pull-in voltage, Young’s modulus, mean stress, CMOS-MEMS

## Abstract

This paper develops the technologies of mechanical characterization of CMOS-MEMS devices, and presents a robust algorithm for extracting mechanical properties, such as Young’s modulus, and mean stress, through the external electrical circuit behavior of the micro test-key. An approximate analytical solution for the pull-in voltage of bridge-type test-key subjected to electrostatic load and initial stress is derived based on Euler’s beam model and the minimum energy method. Then one can use the aforesaid closed form solution of the pull-in voltage to extract the Young’s modulus and mean stress of the test structures. The test cases include the test-key fabricated by a TSMC 0.18 μm standard CMOS process, and the experimental results refer to Osterberg’s work on the pull-in voltage of single crystal silicone microbridges. The extracted material properties calculated by the present algorithm are valid. Besides, this paper also analyzes the robustness of this algorithm regarding the dimension effects of test-keys. This mechanical properties extracting method is expected to be applicable to the wafer-level testing in micro-device manufacture and compatible with the wafer-level testing in IC industry since the test process is non-destructive.

## 1. Introduction

Due to the excellent development of the complementary metal oxide semiconductor (CMOS) technology, many micro-electromechanical systems (MEMS) devices such as comb-fingers [1], micro-mirrors [2], and resonators [3], the so-called CMOS-MEMS, can be fabricated by standard CMOS processes. The main advantage of CMOS-MEMS is batch production. Apart from the electrical testing of circuits, the MEMS-side still requires the mechanical testing of micro-sensing or -actuating components. However, there is no standard mechanical testing method for CMOS-MEMS devices. Characterization of the mechanical properties of CMOS-MEMS devices is important since their performance depends on the constitutive properties of the thin film made by the CMOS process. It is known that the properties of thin films are different from those of bulk materials, depending on the fabrication process. Moreover, large residual stress may induce failure of the micro-devices and circuits. Therefore, the material properties, such as Young’s modulus and residual stress, should be controlled as early as possible to ensure the repeatability for each device.

The property-extraction methods for large-scale implementation in MEMS fabrication require additional measurement and actuation equipment or complicated test structure designs. These methods are not compatible with IC metrology technologies. From the mechanical viewpoint of MEMS devices, the important thin-film material parameters are Young’s modulus [4–15], residual stress [7,9,15–18], Poisson’s ratio and shear modulus [15], residual strain [8,19], and hardness [20]. Among these mentioned parameters, Young’s modulus and residual stress have attracted the most attention. By using appropriate actuation and measurement techniques, these material properties can be extracted by determining the deformation or dynamic response of the test microstructures subjected to given external loads. Table 1 summarizes six different actuation methods and eight different measurement methods for extracting material properties in MEMS devices.

The electrostatic method employs a bias voltage to deflect the microtest structure downward to the ground plane [4,5,8,21]. The vibration method adopts comb drivers, piezoelectric shakers, or acoustic waves to vibrate the microtest structure [6,22]. Pulsed laser light can also be used to excite micro beams [23,24]. The force/pressure method uses the probe of atomic force microscope(AFM) or a nanoindenter to apply a force on the micro test structure or apply barometric pressure on the test membrane [12,13]. The thermal method heats the test structure to deform it [15], yet the heating time, and the uniform temperature field are critical issues in this testing method. The pre-deformation method does not need any actuation as it makes use of the deformation induced by large initial stresses [16,17,19,25]. In [16] the detection film on a cantilever beam firstneeds to be deposited, and then the variance of curvature is observedto determine the residual stress. Interferometers are a very common apparatus for measuring deformations or vibrations. For example, in some literatures [4–6] the optical signal or dynamic response is detected to extract the mechanical properties of test structures. These methods are manual, and need operators to judge the output signal. AFMs and scanning electron microscopes (SEMs) can also be used to measure the deformation. In [18] the difference of test beam length due to the residual stress effect is measured, but the detection signal is too small to identify the strain, therefore a specific apparatus is needed to enlarge the output signal. In some literature X-Ray diffraction (XRD) is used to measure the indentation imprint [14,20]. The pull-in method detects the pull-in voltage of micro test structures [9,26,27]. Besides, a micro tensile test [10] is used to determine the Young’s modulus, but the test samples need to be fabricated in specific shape to fit the clamping apparatus of the tensile tester. Nanoindenters [28] are also a common technique for extracting Young’s modulus and hardness of thin films. Nevertheless, this method can’t extract the residual stress of thin films. The modified Stoney’s equation (Equation (1)) [29] to evaluate the film stress is proposed as follows:

(1) $$\sigma =\frac{{\mathrm{Eh}}^{3}}{6\left(1-\nu \right){\mathrm{Rt}}^{2}\left(1+\frac{t}{h}\right)}$$

where the *σ* is the residual stress of thin film, E/(1 − ν) is the biaxial modulus, *h* is the thickness of the substrate, *t* is the thickness of thin film, and *R* is the radius of curvature of substrate. However, this method needs to determine the biaxial modulus of the thin film first, and it is hard to deal with the case of local areas with extreme variation of radius of curvature. Among these aforementioned techniques, the most common test structures are beams and diaphragms. Ring-type test structures [15] have also been reported, but their underlying fundamental principles are very complicated and they are difficult to fabricate. A viable test method “must be usable at the wafer level in a manufacturing environment, require only readily available test equipment, and it should be supported with documented structure-design, data-acquisition and data-analysis methods, and calibrated models for quantitative interpretation of results” [9]. Out of the known methods, the best candidate for meeting the aforementioned requirements was judged to be the measurement of the electric-circuit behavior of the microstructures subjected to electrostatic loads. Compared to the prior art correlated with complicated or even empirical manipulation of numerical means, this paper builds simple and valid approximate analytical models of the CMOS-MEMS test-keys for extracting mechanical properties. These properties, such as Young’s modulus, and mean stress, are investigated, through the external electrical circuit behavior of the CMOS-MEMS test-keys.

## 2. Electromechanical Behavior of the CMOS-MEMS Bridge Test-key

**Figure 1.**
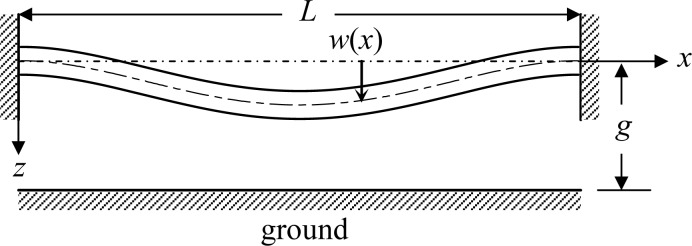
Schematic of the micro fixed-fixed beam.

A conceptual diagram of a micro bridge is shown in Figure 1. The beam is of length *L*, width *b*, thickness *h*, and is separated from the ground by an initial gap *g*. As actuated by a constant drive voltage *V*, the electrostatic force causes a position-dependent deflection *w*(*x*). The following assumptions are made to simulate the bridge:

1. The bridge is homogeneous and with uniform cross section.
2. The bridge is within the Euler-Bernoulli model.
3. The stress gradient is neglected.
4. Small deflection and ideal fixed boundary conditions.

### 2.1. Energy Expression

The mechanical strain energy of an infinitesimal beam element is:

(2) $${\mathrm{dU}}_{m}=\sigma_{0}\left[\frac{1}{2}{\left(\frac{\mathrm{dw}}{\mathrm{dx}}\right)}^{2}\right]d\nu +E\left[\frac{1}{2}z^{2}{\left(-\frac{d^{2}w}{{\mathrm{dx}}^{2}}\right)}^{2}\right]d\nu$$

The total mechanical strain energy of the beam, as shown in Figure 1, can be expressed as:

(3) $$\begin{array}{ll} U_{m} & =\int_{0}^{L}\sigma_{0}\left[\frac{1}{2}{\left(\frac{\mathrm{dw}}{\mathrm{dx}}\right)}^{2}\right]\mathrm{hbdx}+\int_{0}^{L}\mathrm{EI}\left[\frac{1}{2}{\left(-\frac{d^{2}w}{{\mathrm{dx}}^{2}}\right)}^{2}\right]\mathrm{dx}\\ & =\int_{0}^{L}\left[\frac{\sigma_{0}\mathrm{bh}}{2}{\left(\frac{\mathrm{dw}}{\mathrm{dx}}\right)}^{2}+\frac{\mathrm{EI}}{2}{\left(\frac{d^{2}w}{{\mathrm{dx}}^{2}}\right)}^{2}\right]\mathrm{dx} \end{array}$$

where *b*, *E*, *h*, *I*, *L*, and *w* represent the beam width, Young’s modulus, thickness, area inertia moment of beam cross section, beam length, and deflection, respectively. In the integrand of Equation (3), the first term is the strain energy induced by initial stress (*σ*_0_) and the second term is the bending strain energy induced by external loads. The fringing fields are considerable and must be taken into account when modeling the electrostatic loads. For an infinitesimal beam element with length *dx*, the differential capacitance *dC* is given as [36]:

(4) $$\mathrm{dC}=ɛ\left[\left(\frac{b}{g-w}\right)-1.06+3.31{\left(\frac{h}{g-w}\right)}^{0.23}+0.73{\left(\frac{b}{h}\right)}^{0.23}\right]\mathrm{dx}$$

where *ε* and *g* represent the permittivity of dielectric medium and the initial gap between test beam and ground plane, respectively. Hence, the total electrical potential energy *U_e_* is given by:

(5) $$U_{e}=-\int_{0}^{L}\frac{1}{2}{ɛV}^{2}\left[\left(\frac{b}{g-w}\right)-1.06+3.31{\left(\frac{h}{g-w}\right)}^{0.23}+0.73{\left(\frac{b}{h}\right)}^{0.23}\right]\mathrm{dx}$$

where *V* is the applied bias voltage. In Equation (5), the first term is ideal flat plate capacitance, the second term is a length-dependent adjustment parameter, and the third term is the fringing field capacitance due to beam thickness. Then, the total system energy *U* equals the sum of mechanical strain energy and electrical potential energy, *i.e.*,

(6) $$\begin{array}{ll} U & =\int_{0}^{L}\left[\frac{\sigma_{0}\mathrm{bh}}{2}{\left(\frac{\mathrm{dw}}{\mathrm{dx}}\right)}^{2}+\frac{\mathrm{EI}}{2}{\left(\frac{d^{2}w}{{\mathrm{dx}}^{2}}\right)}^{2}\right]\mathrm{dx}\\ & -\int_{0}^{L}\frac{1}{2}{ɛV}^{2}\left[\left(\frac{b}{g-w}\right)-1.06+3.31{\left(\frac{h}{g-w}\right)}^{0.23}+0.73{\left(\frac{b}{h}\right)}^{0.23}\right]\mathrm{dx} \end{array}$$

It should be mentioned that nonlinearities are simplified with only in the electrostatic part of the model. Indeed, the beam structure is assumed linearly elastic, without any consideration of geometrical nonlinearity in virtue of large deformation. Expanding the electrostatic terms in Equation (6) by Taylor’s series with respect to the initial equilibrium position, *i.e.*, *w* = 0, and truncate the fifth and higher order terms since (*w/g*)^n^ << 1 for *n*≥5. Therefore, the total system potential energy *U* becomes:

(7) $$\begin{array}{ll} U & =\int_{0}^{L}\frac{\sigma_{0}\mathrm{bh}}{2}{\left(\frac{\mathrm{dw}}{\mathrm{dx}}\right)}^{2}\mathrm{dx}+\int_{0}^{L}\frac{\mathrm{EI}}{2}{\left(\frac{d^{2}w}{{\mathrm{dx}}^{2}}\right)}^{2}\mathrm{dx}\\ & -\int_{0}^{L}\frac{{ɛV}^{2}b}{2g}\left(1+\left(\frac{w}{g}\right)+{\left(\frac{w}{g}\right)}^{2}+{\left(\frac{w}{g}\right)}^{3}+{\left(\frac{w}{g}\right)}^{4}\right)\mathrm{dx}+\int_{0}^{L}\frac{1.06ɛV^{2}}{2}\mathrm{dx}\\ & -\int_{0}^{L}\frac{3.31ɛV^{2}h^{0.23}}{2g^{0.23}}\left(1+0.23\left(\frac{w}{g}\right)+\frac{0.283}{2}{\left(\frac{w}{g}\right)}^{2}+\frac{0.631}{6}{\left(\frac{w}{g}\right)}^{2}+\frac{2.038}{24}{\left(\frac{w}{g}\right)}^{4}\right)\mathrm{dx}\\ & -\int_{0}^{L}\frac{0.73{ɛV}^{2}}{2}{\left(\frac{b}{h}\right)}^{0.23}\mathrm{dx} \end{array}$$

### 2.2. Approximate Analytical Solution to Pull-in Voltage

The exact solution for the electrostatic-actuated beam is difficult to obtain since it is a nonlinear system with the nonlinear electrostatic force coupled with the structural deflection. Thus, such problem is often solved by the approximate analytical solution. Using the assumed mode method [38], the deflection function *w*(*x*) is expressed as:

(8) $$w\left(x\right)=\sum_{i=1}^{n}\eta_{i}\phi_{i}\left(x\right)$$

where *ϕ_i_*(*x*) is the *ith* mode and the coefficient *η_i_* to be solved is the associated modal participation factors. Then substituting the assumed deflection function into the system energy expression, one can solve for the coefficient *η_i_*. Since the natural mode is the exact solution to the free vibration of structures, it essentially satisfies the boundary conditions and the homogeneous part of the governing equation of a dynamic system. Thus, the natural modes form the foundation for forced response calculations in structural dynamics [38]. The first natural mode of a fixed-fixed beam is adopted since the electrostatic loads are attractive forces and the deflection is much similar to the first natural mode of fixed-fixed beam. The first natural mode of a fixed-fixed beam is [38]:

(9) ϕ(x)=(coshλx−cosλx)−ζ(sinhλx−sinλx)

where the coefficients *ζ* and *λ* satisfy the following equations:

(10) $$\zeta =\frac{\text{cosh}\left(\lambda L\right)-\text{cos}\left(\lambda L\right)}{\text{sinh}\left(\lambda L\right)-\text{sin}\left(\lambda L\right)},\;\;\text{and}\;\;\text{cos}\left(\lambda L\right)\cdot \text{cosh}\left(\lambda L\right)-1=0$$

Substituting Equations (8) and (9) into Equation (7) yields:

(11) $$\begin{array}{ll} U & =\int_{0}^{L}\frac{\sigma_{0}\mathrm{bh}}{2}{\left(\eta \phi^{\prime }\right)}^{2}\mathrm{dx}+\int_{0}^{L}\frac{\mathrm{EI}}{2}{\left(\eta \phi^{\prime \prime }\right)}^{2}\mathrm{dx}\\ & -\int_{0}^{L}\frac{{ɛV}^{2}b}{2g}\left(1+\left(\frac{\eta \phi }{g}\right)+{\left(\frac{\eta \phi }{g}\right)}^{2}+{\left(\frac{\eta \phi }{g}\right)}^{3}+{\left(\frac{\eta \phi }{g}\right)}^{4}\right)\mathrm{dx}+\int_{0}^{L}\frac{1.06{ɛV}^{2}}{2}\mathrm{dx}\\ & -\int_{0}^{L}\frac{3.31{ɛV}^{2}h^{0.23}}{2g^{0.23}}\left(1+0.23\left(\frac{\eta \phi }{g}\right)+\frac{0.283}{2}{\left(\frac{\eta \phi }{g}\right)}^{2}+\frac{0.631}{6}{\left(\frac{\eta \phi }{g}\right)}^{3}+\frac{2.038}{24}{\left(\frac{\eta \phi }{g}\right)}^{4}\right)\mathrm{dx}\\ & -\int_{0}^{L}\frac{0.73{ɛV}^{2}}{2}{\left(\frac{b}{h}\right)}^{0.23}\mathrm{dx} \end{array}$$

The system is in static equilibrium when the first-order derivative of the total potential energy *U* with respect to the coefficient *η* equals zero, *i.e.*, *dU*/*dη* = 0, then one have:

(12) $$\eta \left[\sigma_{0}\int_{0}^{L}\mathrm{bh}{\left(\phi^{\prime }\right)}^{2}\mathrm{dx}+E\int_{0}^{L}I{\left(\phi^{\prime \prime }\right)}^{2}\mathrm{dx}\right]=\frac{{ɛV}^{2}}{2}\left(c_{0}+c_{1}\eta +c_{2}\eta^{2}+c_{3}\eta^{3}\right)$$

Where *c_j_* (j = 0–3) are shown as below:

(13) $$\left\{ \begin{array}{l} c_{0}=\int_{0}^{L}\left({b/g}^{2}+0.76h^{0.23}/g^{1.23}\right)\phi dx\\ c_{1}=\int_{0}^{L}\left(2b/g^{3}+0.94h^{0.23}/g^{2.23}\right)\phi^{2}dx\\ c_{2}=\int_{0}^{L}\left(3b/g^{4}+1.04h^{0.23}/g^{3.23}\right)\phi^{3}dx\\ c_{3}=\int_{0}^{L}\left(4b/g^{5}+1.12h^{0.23}/g^{4.23}\right)\phi^{4}dx \end{array}\right.$$

The coefficients *c_j_* depend only on the geometrical parameters of beam. Whether the equilibrium is stable or unstable is determined by the second-order derivative of the total potential energy with respect to *η*. At the transition from a stable to an unstable equilibrium, the second-order derivatives of the total potential energy with respect to *η* also equals zero, *i.e.*, *d*^2^*U*/*dη*^2^ = 0, then one has:

(14) $$\left[\sigma_{0}\int_{0}^{L}\mathrm{bh}{\left(\phi^{\prime }\right)}^{2}\mathrm{dx}+E\int_{0}^{L}I{\left(\phi^{\prime \prime }\right)}^{2}\mathrm{dx}\right]=\frac{{ɛV}^{2}}{2}\left(c_{1}+{2c}_{2}\eta +{3c}_{3}\eta^{2}\right)$$

Substituting Equation (14) into Equation (12) gives:

(15) $${2c}_{3}\eta^{3}+c_{2}\eta^{2}-c_{0}=0$$

Equation (15) is a cubic equation of *η* and can be solved by Cardan solution[39]. The real number root of Equation (15) gives rise to the coefficient *η_PI_* at pull-in as:

(16) $$\begin{array}{ll} \eta_{\mathrm{PI}} & =\sqrt[{3}]{\left[\left(\frac{1}{4}\frac{c_{0}}{c_{3}}\right)-{\left(\frac{1}{6}\frac{c_{2}}{c_{3}}\right)}^{3}\right]+\sqrt{{\left[\left(\frac{1}{4}\frac{c_{0}}{c_{3}}\right)-{\left(\frac{1}{6}\frac{c_{2}}{c_{3}}\right)}^{3}\right]}^{2}-8{\left(\frac{1}{6}\frac{c_{2}}{c_{3}}\right)}^{6}}}\\ & +\sqrt[{3}]{\left[\left(\frac{1}{4}\frac{c_{0}}{c_{3}}\right)-{\left(\frac{1}{6}\frac{c_{2}}{c_{3}}\right)}^{3}\right]-\sqrt{{\left[\left(\frac{1}{4}\frac{c_{0}}{c_{3}}\right)-{\left(\frac{1}{6}\frac{c_{2}}{c_{3}}\right)}^{3}\right]}^{2}-8{\left(\frac{1}{6}\frac{c_{2}}{c_{3}}\right)}^{6}}}-\left(\frac{1}{6}\frac{c_{2}}{c_{3}}\right) \end{array}$$

Substituting Equation (16) into Equation (14) gives the approximate analytical solution to the pull-in voltage *V_PI_* as:

(17) $$V_{\mathrm{PI}}^{2}=\frac{\sigma_{0}\int_{0}^{L}2\mathrm{bh}{\left(\phi^{\prime }\right)}^{2}\mathrm{dx}+E\int_{0}^{L}2I{\left(\phi^{\prime \prime }\right)}^{2}\mathrm{dx}}{ɛ\left(c_{1}+{2c}_{2}\eta_{\mathrm{PI}}+3c_{3}\eta_{\mathrm{PI}}^{2}\right)}$$

As shown in Equation (17), the pull-in voltage contains two terms, the first one is dependent on initial residual stress, and the second one is dependent on beam flexibility. The pull-in voltage increases as the increasing of initial stress (*σ*_0_) or Young’s modulus (*E*). A beam is considered as wide beam as *b/h*≥ 5. Wide beams exhibit plane strain conditions; therefore, the Young’s modulus (*E*) should be replaced by the equivalent Young’s modulus *Ẽ* = *E*/(1 –*ν*^2^) and the residual stress (*σ*_0_) should be replaced by the equivalent residual stress *σ̃*_0_= *σ*_0_(1 –*ν*^2^). Therefore, Equation (17) yields:

(18) $$V_{\mathrm{PI}}^{2}=\frac{{\tilde{\sigma }}_{0}\int_{0}^{L}2\mathrm{bh}{\left(\phi^{\prime }\right)}^{2}\mathrm{dx}+\tilde{E}\int_{0}^{L}2I{\left(\phi^{\prime \prime }\right)}^{2}\mathrm{dx}}{ɛ\left(c_{1}+2c_{2}\eta_{\mathrm{PI}}+3c_{3}\eta_{\mathrm{PI}}^{2}\right)}$$

## 3. Wafer-Level Mechanical Properties Extracting

### 3.1. Algorithm

The correlation between the pull-in voltage and the material parameters must be formulated quantitatively to realize the idea of extracting mechanical properties from pull-in voltage of the test beam. An equilibrium equation has been derived based on Euler-Bernoulli beam model and the fringing filed capacitance model. The equilibrium equation, Equation (18) yields:

(19) $$S{\tilde{\sigma }}_{0}+B\tilde{E}=V_{\mathrm{PI}}^{2}$$

where the parameters *S* and *B* depend on the geometrical parameters of micro test beam and are given as:

(20) $$S=\frac{\int_{0}^{L}2\mathrm{bh}{\left(\phi^{\prime }\right)}^{2}\mathrm{dx}}{ɛ\left(c_{1}+2c_{2}\eta_{\mathrm{PI}}+3c_{3}\eta_{\mathrm{PI}}^{2}\right)}$$

(21) $$B=\frac{\int_{0}^{L}2I{\left(\phi^{\prime \prime }\right)}^{2}\mathrm{dx}}{ɛ\left(c_{1}+2c_{2}\eta_{\mathrm{PI}}+3c_{3}\eta_{\mathrm{PI}}^{2}\right)}$$

For a given beam with the pull-in voltage *V_PI_*, there are two unknowns in Equation (19), *i.e.*, *σ̃*_0_ and *Ẽ*. Therefore, one needs two test beams with different length to get the two unknowns. For the two test beams made of the same material, but with different length, they have the same Young’s modulus and mean stress but different pull-in voltages and different *S* and *B* parameters. Then, one has two equations:

(22) $$\left\{ \begin{array}{l} S_{1}{\tilde{\sigma }}_{0}+B_{1}\tilde{E}=V_{\mathrm{PI}1}^{2}\\ S_{2}{\tilde{\sigma }}_{0}+B_{2}\tilde{E}=V_{\mathrm{PI}2}^{2} \end{array}\right.$$

By rearranging Equation (22), the mean stress (*σ̃*_0_) and Young’s modulus (*Ẽ*) are given as the following matrix operational form:

(23) $$\left\{\begin{array}{l} {\tilde{\sigma }}_{0}\\ \tilde{E} \end{array}\right\}={\left[\begin{array}{cc} S_{1} & B_{1}\\ S_{2} & B_{2} \end{array}\right]}^{-1}\left\{\begin{array}{l} V_{\mathrm{PI}1}^{2}\\ V_{\mathrm{PI}2}^{2} \end{array}\right\}$$

One can extract Young’s modulus (*Ẽ*) and mean stress (*σ̃*_0_) easily by substituting the measured pull-in voltages of the two test beams with different length into Equation (23).

### 3.2. Algorithm Verification

Osterberg [9] had measured pull-in voltages of numerous fixed-fixed beams with different lengths. The authors selected Osterberg’s measured data of two arbitrary test beams and substituted them into the algorithm to verify the validity of the present method. Table 2 lists the geometrical parameters and pull-in voltages of the selected fixed-fixed beams which are made of mono-crystalline silicon. There are two groups of fixed-fixed beams listed in Table 2; each group contains six beams of different lengths. The difference of the two groups is only the crystalline plane of cross section. The first group is in the (100) crystalline plane while the second one is in the (110) crystalline plane. The author selected two beams from each group and substituted the measured data and beam dimensions into Equation (23) to extract Young’s modulus (*Ẽ*) and mean stress (*σ̃*_0_). Note that the cross-section of the beams of group 1 are in the (100) crystalline plane while that of the group 2 are in the (110) crystalline plane. The Young’s modulus of mono-crystalline silicon in (100) and (110) are 138 GPa and 168 GPa, respectively. The mean stresses of the two beam-samples are 10 MPa [9].

Tables 3 and 4 list the extracted Young’s modulus (*Ẽ*) and mean stress (*σ̃*_0_) of mono-crystalline silicon in (100) and (110), respectively. It is shown that the extracted values of the present algorithm agree well with the average extracted value of Osterberg’s results [9], but with better convergence than Osterberg’s algorithm. The variety of standard deviation *V_SD_* of the extracted Young’s modulus (*Ẽ*) are all within 1% which are almost tenth of the deviations of Osterberg’s results [9] for both mono-crystalline silicon in (100) and (110). Besides, the *V_SD_* of the extracted mean stress (*σ̃*_0_) are all within 2%, which are also almost tenth of the deviations of Osterberg’s results [9] for both mono-crystalline silicon in (100) and (110).

## 4. Experimental Methodology

### 4.1. Sample Preparation

**Figure 2.**
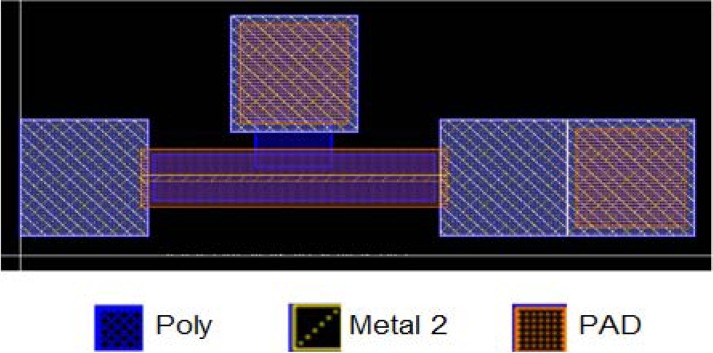
Layout of the bridge-type test-key.

This paper takes bridge-type structures as test structures. The test structures are fabricated by a TSMC 0.18 μm 1P6M standard CMOS process. The upper electrode is metal 2, and the bottom electrode is a poly layer. The anchors are composed of metal and poly layers where viasconnect columns between each metal layer, and contact is the connecting column between metal 1 and the poly layer. When a driving voltage is applied between the test structure and ground plane, the test structure will deflect downward to the ground and this results in a capacitance variation. Figure 2 reveals the layout of the bridge-type test-key. There are two places which are defined as PAD layers-probing pad location and etching hole. The defined area of PAD layer at anchors is the probing pad location. It should be noticed that the probing location must maintain an appropriate distance away from the test structure to avoid measurement uncertainty. The other defined area of PAD layer between the neighboring passivation layers is the etching hole which makes the sacrificial layer etched by etching solution and then results in the structure release. Since the anchors are composed of metal layer, poly layers, via and contact columns, it can reduce the under-cut effect compared to the anchors composed of metal and poly layer without via and contact columns between that when the structures are soaked in etching solution.

**Figure 3.**
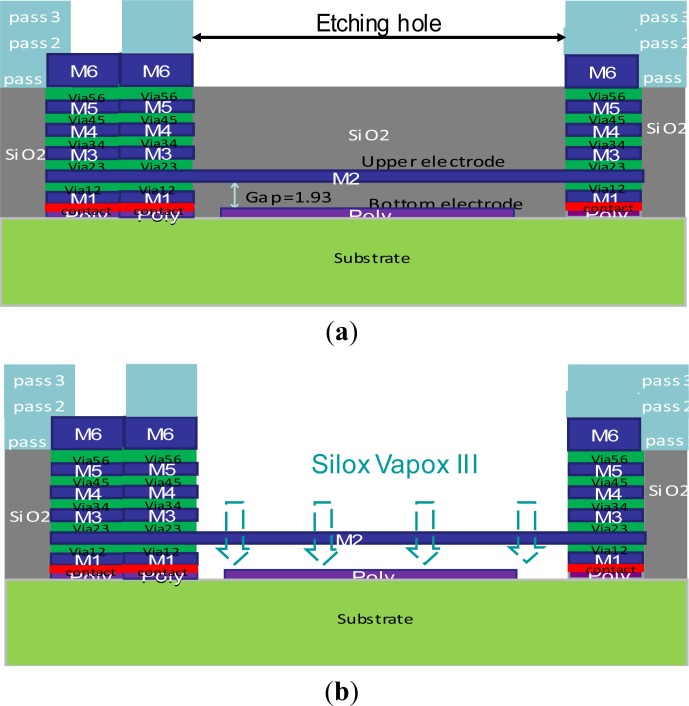
Scheme cross-section of the bridge-type testkey, (**a**) after the CMOS process; (**b**) after post-process.

Figure 3 shows the schematic cross-section of the bridge-type test-key after the CMOS process and after post-process. A silicon dioxide layer between the upper electrode and the bottom electrode is a sacrificial layer (Figure 3(a)). Silox Vapox III is used as the etching solution since it has good etch rate selectivity between the metal and silicon dioxide layer. Soaking the bridge-type test-key after the CMOS process in Silox Vapox III, the etching solution will etch the silicon dioxide layer through the etching hole between the neighboring passivation layers, and form a gap between both electrodes (Figure 3(b)) during post-processing. Next, we utilize the critical point drying (CPD) method to dry the structures without collapsing to release the structures. It should be mentioned that the size of the etching hole affects the release time, and the appropriate design of the etching hole can make the release time shorter. Besides, setting a magnetic stirring rod below the etching solution tank can also shorten the release time, but one must make sure the structures have a protective pattern to prevent the damage caused during the stirring process. Excepting the probing pad location and etching hole, the other places are covered by passivation layers to avoid the damage caused by the etching solution.

**Figure 4.**
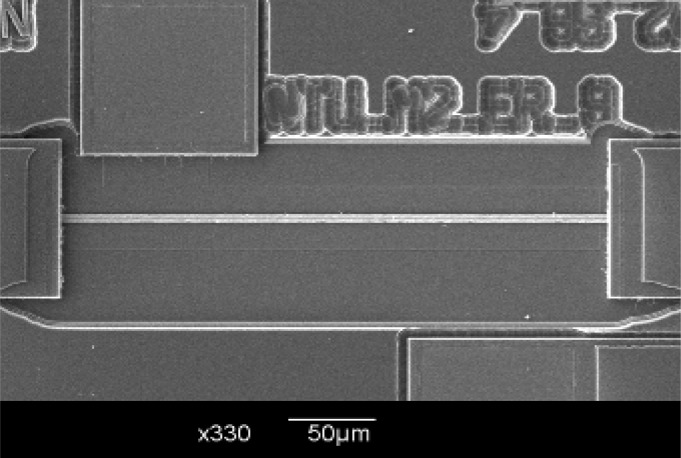
SEM picture of the bridge-type test-key after post-processing.

Figure 4 shows SEM pictures of the bridge-type test-key after post-processing, and it is obvious that the test-key is released successfully by soaking in Silox Vapox III for 85 min.

### 4.2. Pull-in Voltage Detecting

**Figure 5.**
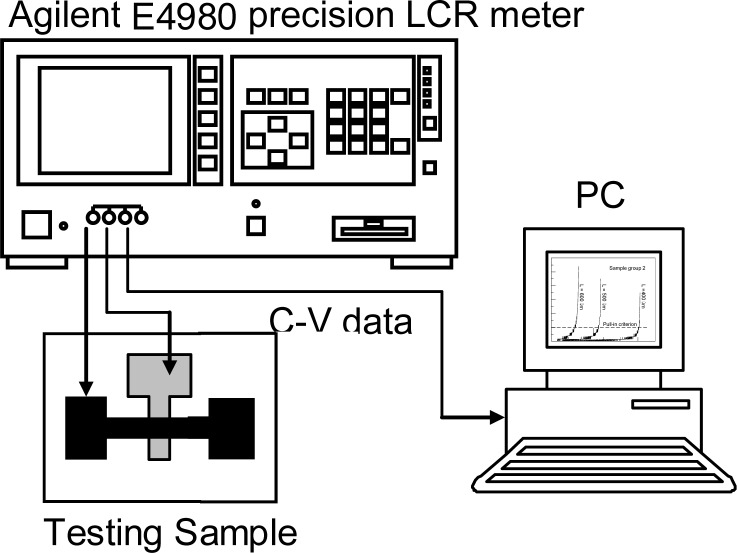
Schematic of the experiment setup for pull-in voltage detection.

For a deflective microstructure subjected to electrostatic loads, as shown in Figure 1, the structural deflection causes a change in the gap between the upper and bottom electrodes and thus a change in the capacitance. Therefore, the variation of capacitances with the applied bias voltages is equivalent to the deformations to the external loads, and then one can detect the pull-in voltage by tracking the capacitance sensitivities with respect to applied bias voltages. Pull-in will occur when the capacitance shows a sharp increase. Through the capacitance-voltage measurement and the material property extraction algorithms mentioned above, one can obtain the material properties of the test microstructure. The principle of capacitance-voltage measurement is introduced as the following. The main idea is to measure the circuit capacitive reactance *X_C_* to yield the capacitance *C*. The capacitive reactance is *X_c_* = 1/(2*π·f·C*) where *X_C_*, *f*, and *C* represent the circuit capacitive reactance, the testing signal frequency, and the capacitance, respectively. The input driving voltage is a small AC testing signal riding on a large DC bias voltage which induces the structural deflection. Then the capacitance can be calculated from the circuit capacitive reactance. It should be mentioned here that the frequency of the AC testing signal must avoid the resonance frequency of the test microstructure; otherwise the capacitance will show a large fluctuation. The Agilent E4980 precision LCR meter is used to measure the capacitance-voltage (C-V) variation of the test microstructure, as shown in Figure 5.

The frequency and level of the AC testing signal must be set properly since they will affect the accuracy of the capacitance measurement. The authors chose the root mean square value of the test AC signal level as 25 mV and the frequency 1 MHz. The integration time is set to medium (MED). The instrument parameters setting are listed in Table 5.

**Figure 6.**
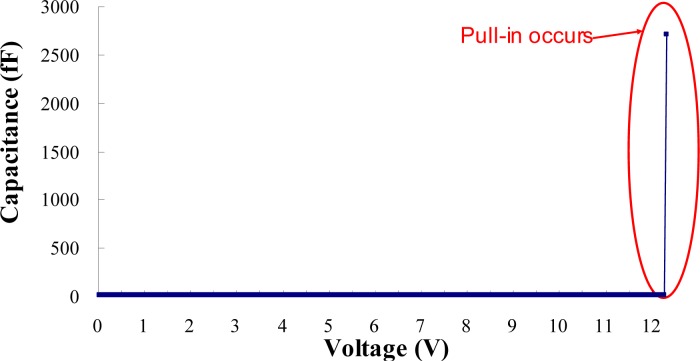
Typical sensitivities curves of the capacitances with respect to applied bias voltages of the test beam.

The pull-in voltage is detected by tracking the capacitance sensitivities with respect to the applied bias voltages. Two low noise probes touch the two probing pad of test beam, as shown in Figure 5. The probes are connected to the Agilent E4980 high precision LCR meter, which can supply a test signal of 25 mV/1 MHz riding on the bias voltages ranging from 0 to 40 V. Agilent E4980 exports the capacitance-voltage data to a personal computer and tracks the capacitance sensitivities to the applied bias voltage. Figure 6 shows the typical measured capacitance sensitivities results. Pull-in will occur when the capacitance is with sharp increase. Therefore, according to the results from capacitance-voltage measurement, one can obtain the pull-in voltage of the test beam exactly.

Table 6 lists the geometrical parameters of the bridge-type test beams which are fabricated by a TSMC 0.18 μm 1P6M standard CMOS process. The test beams have the same width, gap, and thickness, but different length. One knows that pull-in occurs when capacitance increases sharply. According to Figure 6, it is obvious that the capacitance will rise up to ten fold or even hundred fold compared to the original capacitance when pull-in occurs. Therefore, one can get the pull-in voltage of test beams, and the corresponding data is shown in Table 7, where *V_PI-ave_* is the average value of measured pull-in voltage for five times of each test beam, and Δ*V_PI_* is the corresponding standard deviation.

## 5. Results and Discussion

We substitute the experimental results in Table 7 into Equation (23) to extract Young’s modulus (*Ẽ*) and mean stress (*σ̃*_0_). Table 8 shows the extracted Young’s modulus (*Ẽ*) and mean stress (*σ̃*_0_) of the two test beams when the length difference (Δ*L*) equals 50 μm. The extracted Young’s modulus (*Ẽ*) and mean stress (*σ̃*_0_) are 132.01 ± 13.48 GPa and 3.4 ± 0.15 MPa, respectively.

This work presents an algorithm for extracting Young’s modulus (*Ẽ*) and mean stress (*σ̃*_0_) of structural materials of CMOS-MEMS devices by detecting the pull-in voltages of two micro bridge-type test beams. The overall deviations of the extracted Young’s modulus (*Ẽ*) and mean stress (*σ̃*_0_) of the demonstrated materials are within 11% and 5%, respectively, when the two test beams have a length difference (Δ*L*) equal to 50 μm. The present method is very suitable for the implementation of the mechanical characterization of capacitive CMOS-MEMS devices in wafer level testing. The present algorithm can easily be written as a programming code and accompanied by an LCR meter to realize the wafer-level testing for CMOS-MEMS manufacture.

**Figure 7.**
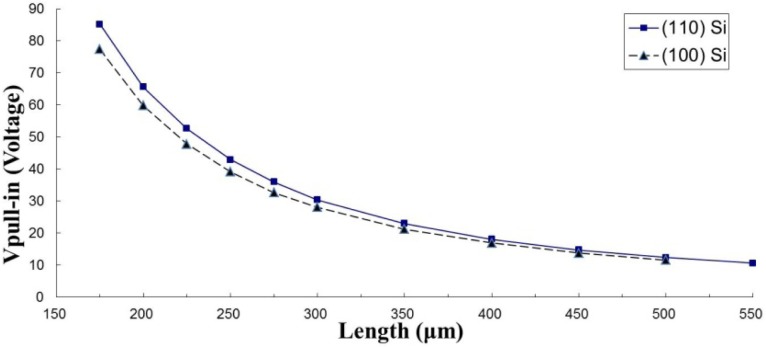
The measured the pull-in voltages of the fixed-fixed beams made of mono-crystalline siliconin (100) and (110) orientations in Osterberg’s work [9].

**Figure 8.**
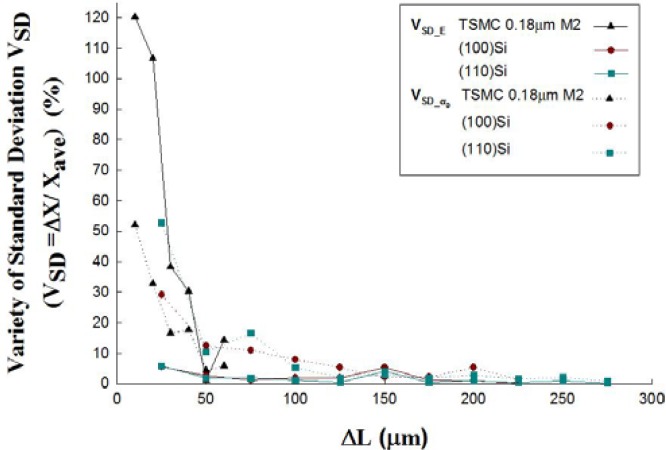
The variation of the extracted values by this work.

Since this testing method needs to measure pull-in voltages of two test beams with different length, attention should be paid to the appropriate length design of the two test beams. Table 2 lists the geometrical parameters and Figure 7 shows the measured pull-in voltages of the fixed-fixed beams which are made of mono-crystalline silicon in published work [9]. The author selected any two beams and substituted the measured data and beam dimensions into Equation (23) to extract the Young’s modulus (*Ẽ*) and mean stress (*σ̃*_0_). The same procedure is used to deal with the experimental results in Table 7, where the test beams are fabricated by a TSMC 0.18 μm 1P6M standard CMOS process. According to the extracted results, the extracted values show small standard deviations for large Δ*L* cases but with a large standard deviation for small Δ*L* cases in two kinds of common structural materials, such as the material made by the TSMC 0.18 μm standard CMOS process, and mono-crystalline silicon in (100) and (110) orientations. The relationship between the difference of test beams (Δ*L*) and the variation of the extracted values (*V_SD_*) by this work is shown in Figure 8. It indicates that the variation of Young’s modulus(*V_SD_E_*) and mean stress (*V_SD__σ_0_*) will reduce by15% when Δ*L* is larger than 50 μm, even being as low as 2% for Δ*L* equal to 225 μm in mono-crystalline silicon testing cases. These evidences show that the algorithm presented in this work is robust in extracting mechanical properties at wafer-level testing when test keys with appropriate length design.

## 6. Conclusions

This paper presents a robust algorithm for extracting Young’s modulus, and mean stress of structural materials of CMOS-MEMS devices. By detecting the pull-in voltages of two bridge-type test beams, and applying the characteristics to the equivalent electromechanical models, and one can know the mechanical properties of thin films. The contributions of this paper may be described in detail as follows:

First, the paper has demonstrated the present method with two common structural materials, such as the material made by the TSMC 0.18 μm standard CMOS process, and mono-crystalline silicon in (100) and (110) orientations. The extracted values by the present method are summarized in Table 9. The overall deviation of the extracted Young’s modulus, mean stress, and gradient stress of the structural materials made by the TSMC 0.18 μm standard CMOS process are within 11% and 5%, respectively. Besides, the deviations of the extracted Young’s modulus and mean stress are within 1% and 2% which are almost tenth of the deviations of Osterberg’s results [9] for mono-crystalline silicon in (100) and (110) orientations. Second, the study of the robustness of the present method with regards to the dimension effects of the test-key is discussed in this paper. For the dimension effects of the test-key, the variations of Young’s modulus (*V_SD_E_*) and mean stress (*V_SD__ σ_0_*) are discussed. According to the results shown in Figure 8, the authors infer that the *V_SD_E_* and *V_SD__ σ_0_*will be reduced within 15% for Δ*L* larger than 50 μm, and even within 2% for Δ*L* larger than 225 μm in mono-crystalline silicon testing cases. Therefore, the authors recommend that the two test beams should have a length difference (*ΔL*) which is larger than 50 μm to decrease the dimension effect. Third, the CMOS-MEMS test-key can be set at the scribe line, and removed after testing. Therefore, it doesn’t need any extra area to proceed with structural material testing. Fourth, the present method is very suitable for the implementation of the mechanical characterization of CMOS-MEMS devices in wafer level testing since the testing signals are electrical signals. Moreover, the present algorithm can easily be written as a programming code and accompanied by an LCR meter to realize wafer-level testing for MEMS manufacture.

## Figures and Tables

**Table 1.** Summary of actuation and measurement methods for extracting material properties.

**Measuring methods**	**Actuating methods**					
	**Electrostatic**	**Vibration**	**Pulsed laser light**	**Force/Pressure**	**Thermal**	**Pre-deformation**
Interferometer	[4,5,8,21,30–32]	[6,22]	[23,24]	[7,10,33]	[15,30–32]	[16,17,19,25]
Nanoindenter				[14,20]		
AFM				[12,13]		
SEM				[11]		
μ strain gauge				[18]		
XRD				[14,20]		
V-F converter	[34]					
Pull-in	[9,26,27,35]					

**Table 2.** Geometrical parameters of the mono-crystalline silicon beam samples and the measured pull-in voltages [9].

**Parameters**	**Values**					
Permeability of free space *ε* (F/m)	8.85 × 10^−12^					
Initial gap *g* (μm)	1.05					
Beam width *b* (μm)	50					
Beam thickness *h* (μm)	2.94					

Length *L* of group 1	175	400	225	450	275	500
	
Measured pull-in voltage *V_PI_* (V)	77.38	16.9	47.79	13.78	32.65	11.56

Length *L* of group 2	175	450	225	500	275	550
	
Measured pull-in voltage *V_PI_* (V)	85.22	14.78	52.68	12.4	36	10.61

**Table 3.** Extracted Young’s modulus and mean stress of the mono-crystalline silicon in (100) crystalline plane and the comparison with Osterberg’s work [9].

**Length (μm)**		**The values extracted by this work**		**M-test [9]**	
*L*_1_	*L*_2_	*E* (GPa)	*σ*_0_ (MPa)	*E* (GPa)	*σ*_0_ (MPa)
175	400	135.35	9.97		
225	450	135.21	9.68	138 ± 4	10 ± 2
275	500	134.40	9.66		
Average (*X_ave_*)		134.99	9.77	138	10
Standard Deviation (Δ*X*)		0.42	0.14	4	2
Variety of Standard Deviation *V_SD_* (*V_SD_* = Δ*X*/*X_ave_*)		0.31%	1.45%	2.90%	20.00%

**Table 4.** Extracted Young’s modulus and mean stress of the mono-crystalline silicon in (110) crystalline plane and the comparison with Osterberg’s work [9].

**Length (μm)**		**The values extracted by this work**		**M-test [9]**	
*L*_1_	*L*_2_	*E* (GPa)	*σ*_0_ (MPa)	*E* (GPa)	*σ*_0_ (MPa)
175	450	166.88	9.50	168 ± 6	10 ± 1
225	500	168.16	9.53		
275	550	167.40	9.69		
Average (*X_ave_*)		167.48	9.57	168	10
Standard Deviation (Δ*X*)		0.53	0.08	6	1
Variety of Standard Deviation *V_SD_* (*V_SD_* =Δ*X*/*X_ave_*)		0.31%	0.87%	3.57%	10.00%

**Table 5.** Measurement conditions.

**Function**	**Cp-D**
Testing Signal Frequency	1 MHz
Testing Signal Level	0.025 V
Bias Voltage Range	0–40 V
Bias Voltage Step	0.05 V
Integration Time	Med

**Table 6.** Geometrical parameters of the bridge-type test beams.

**Parameters**	**Values**
Beam width *b* (μm)	5
Initial gap *g* (μm)	1.93
Beam thickness *h* (μm)	0.53
Beam length *L* (μm)	220−300

**Table 7.** The average and standard deviation of pull-in voltage value of each test beam.

**Length *L* (μm)**	**Vpull-in *V_PI_* (V)**	**Average *V_PI-ave_* (V)**	**Standard Deviation *ΔV_PI_***
220	12.27,12.32,12.32,12.47,12.82	12.44	0.20
230	11.56,11.71,11.76,12.27,12.37	11.93	0.32
240	10.91,11.41,11.46,11.51,11.56	11.36	0.23
250	9.76,10.46,10.71,10.91,11.11	10.59	0.47
260	9.76,10.06,10.16,10.26,10.56	10.16	0.26
270	9.01, 9.11,9.52, 9.71, 9.96	9.46	0.36
280	8.81, 8.86,8.86 ,9.16, 9.51	9.04	0.27
290	8.06, 8.61, 8.86, 8.86,9.51	8.78	0.47
300	8.06 ,8.11, 8.31, 8.56,8.61	8.33	0.22

**Table 8.** Extracted Young’s modulus and mean stress of structural material fabricated by TSMC 0.18 μm 1P6M standard CMOS process.

**Length difference (μm)**	**Length (μm)**		**The extracted values by this work**	
Δ*L*	*L*_1_	*L*_2_	*E* (GPa)	*σ*_0_ (MPa)
50	220	270	112.75	3.56
	230	280	147.87	3.15
	240	290	140.74	3.41
	250	300	126.68	3.48

Average (*X_ave_*)			132.01	3.40
Standard Deviation (Δ*X*)			13.48	0.15
Variety of Standard Deviation *V_SD_* (*V_SD_* =Δ*X*/*X_ave_*)			10.21%	4.52%

**Table 9.** The extracted results for common structural materials.

**Common Structural Material**	**The extracted values by this work**	
metal 2 made by the TSMC 0.18 μm standard CMOS process	*E*(GPa)	*σ*_0_(MPa)
	132.01 ± 13.48	3.4 ± 0.15
mono-crystalline silicon in (100)	134.9 9± 0.42	9.77 ± 0.14
mono-crystalline silicon in (110)	167.48 ± 0.53	9.57 ± 0.08
